# Comparative assessment of differently randomized accelerated particle swarm optimization and squirrel search algorithms for selective harmonics elimination problem

**DOI:** 10.1038/s41598-024-62686-9

**Published:** 2024-06-03

**Authors:** Muhammad Ayyaz Tariq, Muhammad Salman Fakhar, Ghulam Abbas, Syed Abdul Rahman Kashif, Ateeq Ur Rehman, Khmaies Ouahada, Habib Hamam

**Affiliations:** 1 Department of Electrical Engineering, University of Engineering and Technology, Lahore, 54890 Pakistan; 2 Department of Electrical Engineering, The University of Lahore, Lahore, 54000 Pakistan; 3 School of Computing, Gachon University, Seongnam, 13120 Republic of Korea; 4 Department of Electrical and Electronic Engineering Science, School of Electrical Engineering, University of Johannesburg, Johannesburg, 2006 South Africa; 5 Faculty of Engineering, Uni de Moncton, Moncton, NB E1A3E9 Canada; 6 Hodmas University College, Taleh Area, Mogadishu, Somalia; 7 Bridges for Academic Excellence, Centre Ville, Tunis, Tunisia

**Keywords:** Randomization, Accelerated particle swarm optimization (APSO), Squirrel search algorithm (SSA), Statistical analysis, Metaheuristic algorithms, Multilevel inverter (MLI), Types of distributions, Selective harmonics elimination (SHE), Total harmonic distortion (THD), Energy science and technology, Engineering

## Abstract

A random initialization of the search particles is a strong argument in favor of the deployment of nature-inspired metaheuristic algorithms when the knowledge of a good initial guess is lacked. This article analyses the impact of the type of randomization on the working of algorithms and the acquired solutions. In this study, five different types of randomizations are applied to the Accelerated Particle Swarm Optimization (APSO) and Squirrel Search Algorithm (SSA) during the initializations and proceedings of the search particles for selective harmonics elimination (SHE). The types of randomizations include exponential, normal, Rayleigh, uniform, and Weibull characteristics. The statistical analysis shows that the type of randomization does impact the working of optimization algorithms and the fittest value of the objective function.

## Introduction

Selective harmonics elimination (SHE) has been an important problem in electronics regarding multilevel inverters (MLIs). The non-linear loads reduce the power quality with the introduction of harmonics^1^. The parameter for the analysis of harmonics-related problems is total harmonic distortion (THD). MLIs have been deployed in the literature for being the source of better-quality voltage output^2^ and lower voltage gradient in comparison with the square wave inverters^3^. SHE gets rid of the targeted harmonic(s)^4^, reduces the size of the required filter and aids in improving the power quality^5^. Mostly, the lower-order harmonics are eliminated through this process. The quarter-wave symmetry of the narrated inverters aids in simplifying the discussed problem. As more voltage output levels are introduced in the inverter, more orders of harmonics can be eliminated from the system, but the structure and control become complex^6^. As far as the optimization is concerned, a greater number of voltage output levels correspond to more variables to be calculated and hence causing an increment in the dimension of the problem^6^. The variables involved in the optimization problem are enveloped in the positions of the search particles constituting the algorithm. The locations of the search particles convey the firing angles of the switches of the inverters. The firing angles decide the harmonics values, which in turn decide the THD percentage^7^.

The optimization algorithms use a randomization function to spread the search particles during the initialization of the generation. They also deploy a similar concept as an optional constituent of updating the locations during the exploration and exploitation of the search space^8^. One way to improve the performance of algorithms is by introducing different initialization methods. Some effective and robust initialization methods have been suggested in the literature^9–12^; however, the purpose of the presented study is to analyze the significance of the distribution of randomness during initialization and progression of algorithms. Generally, uniform distribution between (0,1) is deployed for this process. Different types of distributions have different ways of spreading the variables between two corner values. Some types of randomizations have shaping and scaling parameters to modify the operational manner^13^ further. Different parameters or functions related to the distributions can be used to ensure that the impact of randomization lies within the specified limits^13^. The accelerated Particle Swarm Optimization (APSO) algorithm has been deployed to solve various problems, including SHE^6^. APSO uses the position and velocity of the search particles along with the randomization to perform optimization^14^. The Squirrel Search Algorithm (SSA) algorithm is an algorithm based on the gliding behavior of flying squirrels^15^ and has been deployed to improve the harmonic profile^16^. Both algorithms are briefly explained to the readers in later sections.

The paper is arranged as follows: the objective function is explained in “Problem description” section, along with the perspective that envelops the upcoming discussion. “Methodology” section covers the methodology of the algorithms and varying distributions. “Results and discussion” section details the trials and their outcomes with the statistical analysis. The article is finally concluded in “Conclusion and Future works” section.

## Problem description

**Figure 1:**
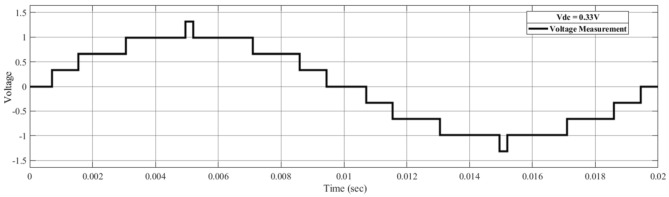
Output voltage waveform of nine-level inverter.

SHE targets the specific harmonic content through the objective function. In this study, THD is considered to be the objective function with the first order set to the desired value and the remaining orders set to zero. The number of targeted contents depends on the number of levels of the inverter and the number of firing angles involved in the problem^6^. Consider Fig. 1 as just an example of the multilevel voltage output for a nine-level inverter.

In Fig. 1, each change in the voltage up to the quarter-wave corresponds to a firing angle (four firing angles in the depicted scenario). As mentioned before, the narrated symmetry simplifies the analysis of the whole cycle. The structure of a cascaded H-bridge circuit is constructed by cascading individual H-bridges^17^. The cascaded H-bridge circuit, equations expressing the harmonic contents and the involved constraint on the firing angles for cascaded H-bridge MLIs are mentioned in^6^. In this study, the objective function deploying those contents is taken to be THD and the successive angle should be at least one degree apart, which is different from the one mentioned in^6^. THD is equated as:

1 $$J = \frac{{\sqrt {\sum\limits_{l = 0}^{k - 1} {V_{2l + 1}^{2} } } }}{{V_{1} }}$$

with

2 $$V_{n} = \sum\limits_{i = 1}^{k} {\cos } (n\alpha_{i} )$$

where *k* is the number of firing angles involved in the respective scenario and α is the firing angle. The value of *k* is equal to 4 in case of 9-level inverter, 5 for 11-level inverter and 6 for 13-level inverter. It also represents the dimension of the problem or the number of unknowns.

The other aspect to be discussed in this section is the type of random distributions while initializing the particles generation and proceeding the algorithms. Uniform randomness follows a uniform spread of the search particles between the imposed limits (0,1). The other discussed types of distributions include exponential, normal, Rayleigh and Weibull. Their parameters are selected so as to keep them between the specified limits (0,1) every time the randomization is deployed. More on this is explained in the next section.

## Methodology

The methodologies involved in this study are APSO and SSA algorithms. The brief discussion regarding these algorithms is as follows.

### Accelerated particle swarm optimization

APSO is based on the birds’ swarming behavior and the main information is hidden in the location of the particle which is updated by taking the influences from randomization, current position and global best positions in an iteration. The single update equation^6^ is:

3 $$x_{i}^{t + 1} = (1 - \beta )x_{i}^{t} + \beta g + \alpha \varepsilon$$

where α and β are constants and $\epsilon$ is a random number between 0 and 1. The variable ${x}_{i}$ is *ith* particle’s current location, $g$ is the global best value, and *t* is the iteration number. The pseudo-code is mentioned in^6^. The global best position value at the end of the process contains the required optimized switching angles.

### Squirrel search algorithm

SSA is based on the dynamic foraging of flying squirrels who glide by lift and drag forces’ modification^18^. They prefer to consume abundantly available acorns during autumn while saving the other nuts for unfavorable weather conditions. Three types of considered trees are: normal tree (with no food), acorn tree and hickory tree. The optimal food source is represented by the location of the hickory tree while the next bests are acorn trees. The updated equation for movement from acorn towards hickory^15^ is:

4 $$x_{at}^{t + 1} = x_{at}^{t} + d \times G \times (x_{ht}^{t} - x_{at}^{t} )$$

where ${x}_{at}^{t}$ is the location of flying squirrel at acorn tree for *tth* iteration, ${x}_{ht}^{t}$ is the location of flying squirrel at hickory tree for *tth* iteration, *d* and *G* are gliding distance and gliding constant respectively. This equation is only applied if a random number is greater than or equal to the predator presence probability^15^, otherwise a random movement is applied. The update equation for movement from normal towards acorn^15^ is:

5 $$x_{nt}^{t + 1} = x_{nt}^{t} + d \times G \times (x_{at}^{t} - x_{nt}^{t} )$$

where ${x}_{nt}^{t}$ is the location of flying squirrel at normal tree for *tth* iteration. This equation is only applied if a random number (not necessarily same as the previous one) is greater than or equal to the predator presence probability, otherwise a random movement is applied. The update equation for movement from normal towards hickory^15^ is:

6 $$x_{nt}^{t + 1} = x_{nt}^{t} + d \times G \times \left( {x_{ht}^{t} - x_{nt}^{t} } \right)$$

**Figure 2:**
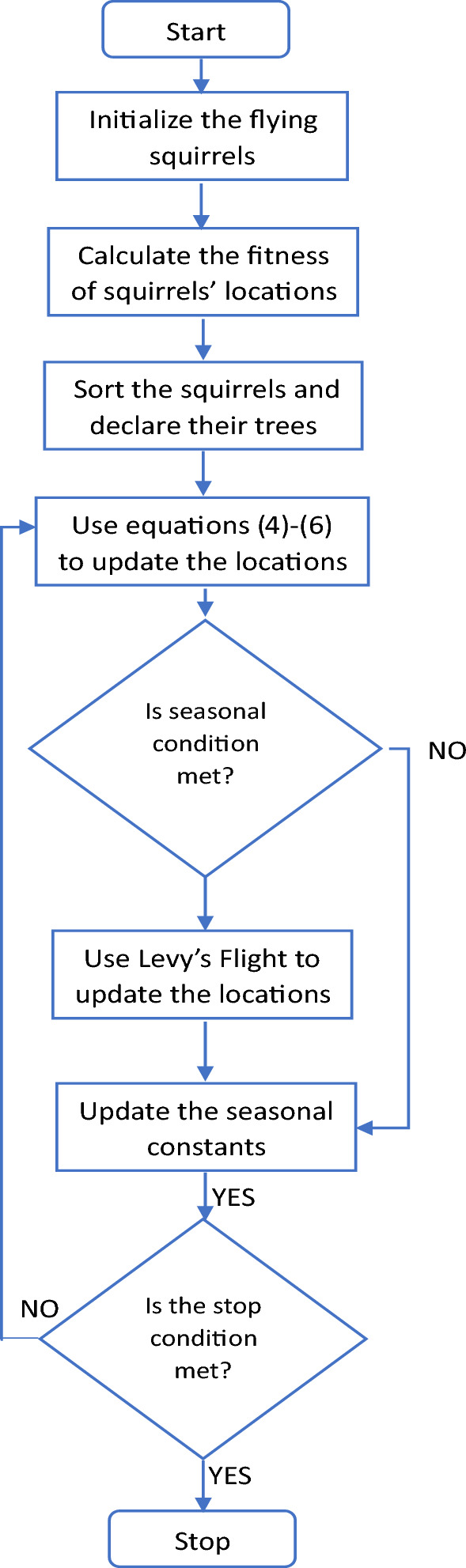
Flowchart of SSA^15^.

This equation is only applied if a random number (not necessarily the same as the previous ones) is greater than or equal to the predator presence probability; otherwise, a random movement is applied. Moreover, Levy flight^19^ is introduced for the squirrels who survived bad seasonal conditions and move to different directions in search of food. Seasonal conditions are evaluated on the basis of seasonal constants which depend on the location of squirrels and the iterations^15^. The flowchart of the algorithm is shown in Fig. 2.

### Types of randomizations

In this study, five types of randomizations have been deployed to exhibit the impact of the nature of randomness. Uniform randomness between (0,1) is obtained by using rand command on MATLAB. Exponential randomness is obtained via conversion of uniform randomness with upper and lower limits of 1 and 0, respectively having λ = 1. Similarly, the normal randomization is attained via conversion of uniform random variable with a mean of 0.5 and sigma of 0.12. A similar process is deployed to attain Rayleigh randomness with sigma of 0.25 and Weibull randomness with shape and scale parameters values of 4.5 and 0.6 respectively. The parameters are of such a choice so as to keep the randomness value between 0 and 1. These randomizations impact the initializations and updates of the positions of the search particles involved in both algorithms. The sample size of the results data is enough to deploy the benefits of the central limit theorem^20^, and thus the SPSS-based ANOVA and independent t-test^21^ are used as standards of statistical comparisons in this study.

## Results and discussion

In this section, the results of different sub-scenarios based on the variations in generation sizes, maximum iterations, and dimensions of the problem are presented and discussed through statistical analysis. The generation size of the search particles and the number of iterations impact the exploration and exploitation of the search space. Moreover, the generation size is vital while dealing with different dimensions of the problem, and the maximum number of iterations decides the count of the re-iteration of randomness along with the progress of algorithms. The details of the results and discussion are as follows:

### 9-level inverter scenario

First, a 9-level inverter scenario is discussed. In this case, the global best search particle contains four firing angles. Both algorithms are run 51 times to find the best firing angles and the lowest cost value under varying sub-scenarios of generation sizes and maximum number of iterations. The first sub-scenario has a generation size of 100 and the maximum iterations equal to 500, the second and third sub-scenarios have the same generation size, but the maximum iterations are 1000 and 2000, respectively. The fourth to sixth sub-scenarios have a generation size of 250 and maximum iterations are kept as 500, 1000, and 2000, respectively. The generation size of 500 and maximum iterations of 500, 1000, and 2000, respectively, constitute the seventh to ninth sub-scenarios. These sizes and iterations constitute nine different sub-scenarios of the 9-level inverter case. The tenth sub-scenario takes generation size of 2000 and maximum iterations equal to 5000. In each sub-scenario, both algorithms are run 51 times for each of the five different randomization techniques.

### 11-level inverter scenario

Secondly, optimization is done for the 11-level inverter-based problem. In this case, the global best search particle contains five firing angles. Both the algorithms are run 51 times to find out the best firing angles and the lowest cost value under ten different sub-scenarios that are already mentioned. In each sub-scenario, both algorithms are tested for five different randomization techniques.

### 13-level inverter scenario

Lastly, a 13-level inverter case is performed. In this case, the global best search particle contains six firing angles. APSO and SSA algorithms with five randomizations are run 51 times each to find out the optimized firing instants and the best objective value for ten different sub-scenarios that are already mentioned.

### Statistical analysis

SPSS is used to analyze the processes on the basis of statistics^21^. Since a sufficient sample size of data is available to deploy the benefit of central limit theorem^20^ and more than two perspectives are to be compared, ANOVA test has been used as the standard to differentiate between the central points of the outcomes produced by the five randomizations in each sub-scenario of every case. In case of lower sample size, Kruskal Wallis test^21^ can be used. The results are presented in tabular as well as in pictorial manner. Moreover, the impact of generation sizes, maximum iterations, and problem dimensions are presented. In this discussion, exponential randomization is denoted by ER, normal by NR, Rayleigh by RR, uniform by UR and Weibull by WR.

**Figure 3:**
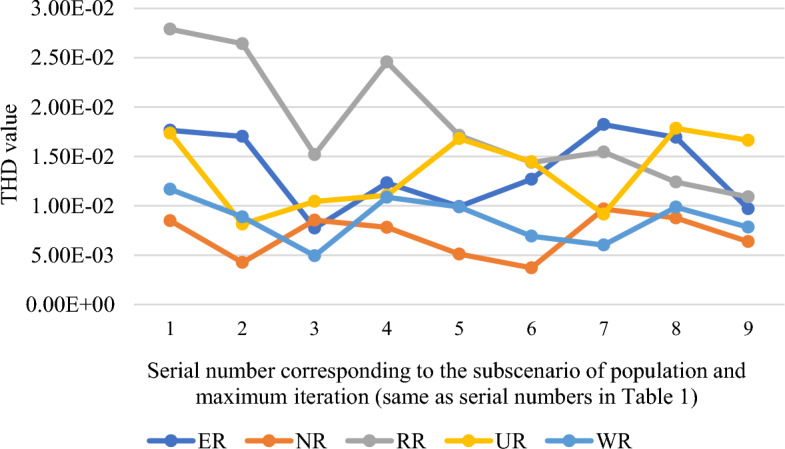
Minimum objective values variation with randomness, population and iterations via SSA for 11-level case.

**Figure 4:**
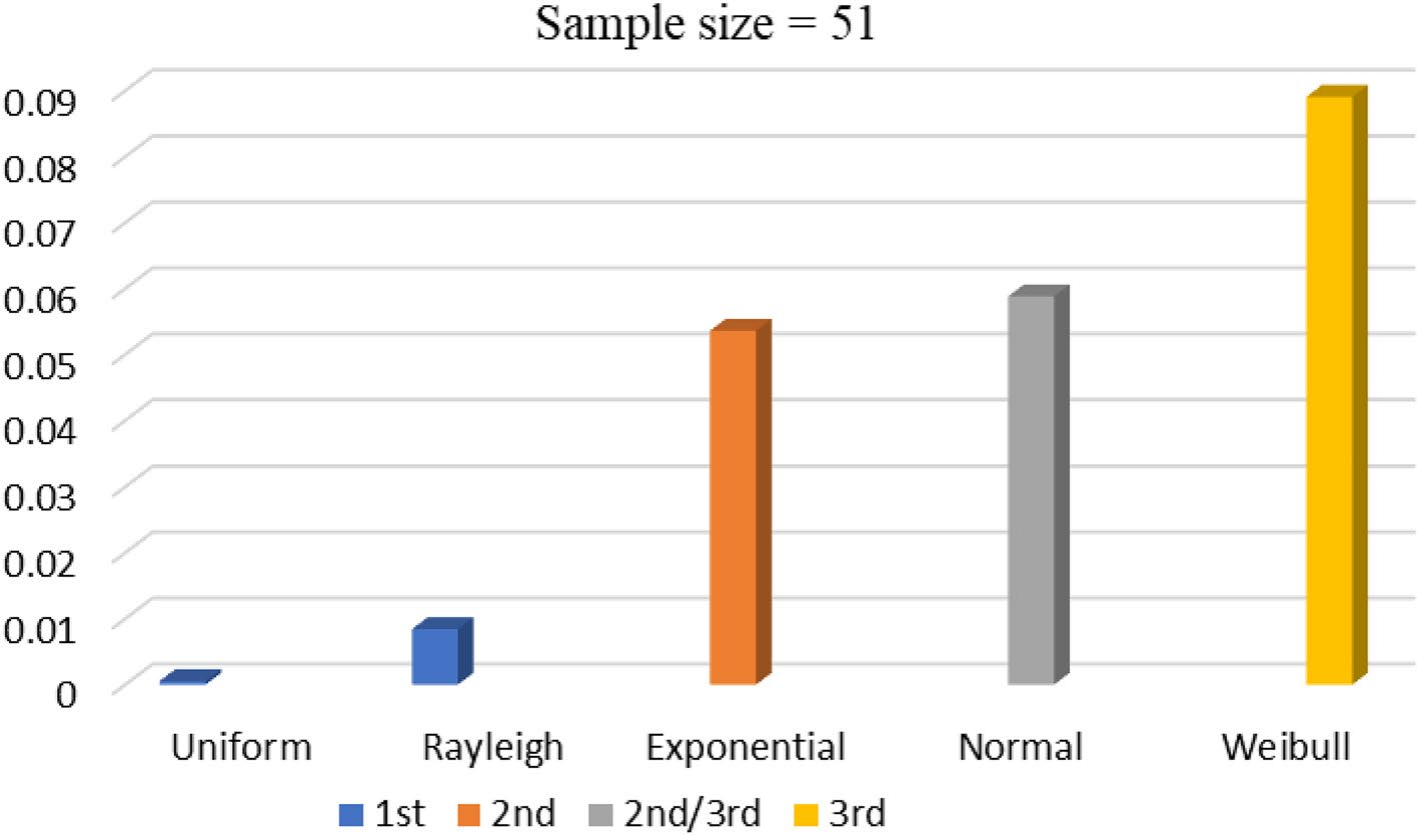
Tukey test results for different randomness via APSO for 11-level case (10th sub-scenario).

**Figure 5:**
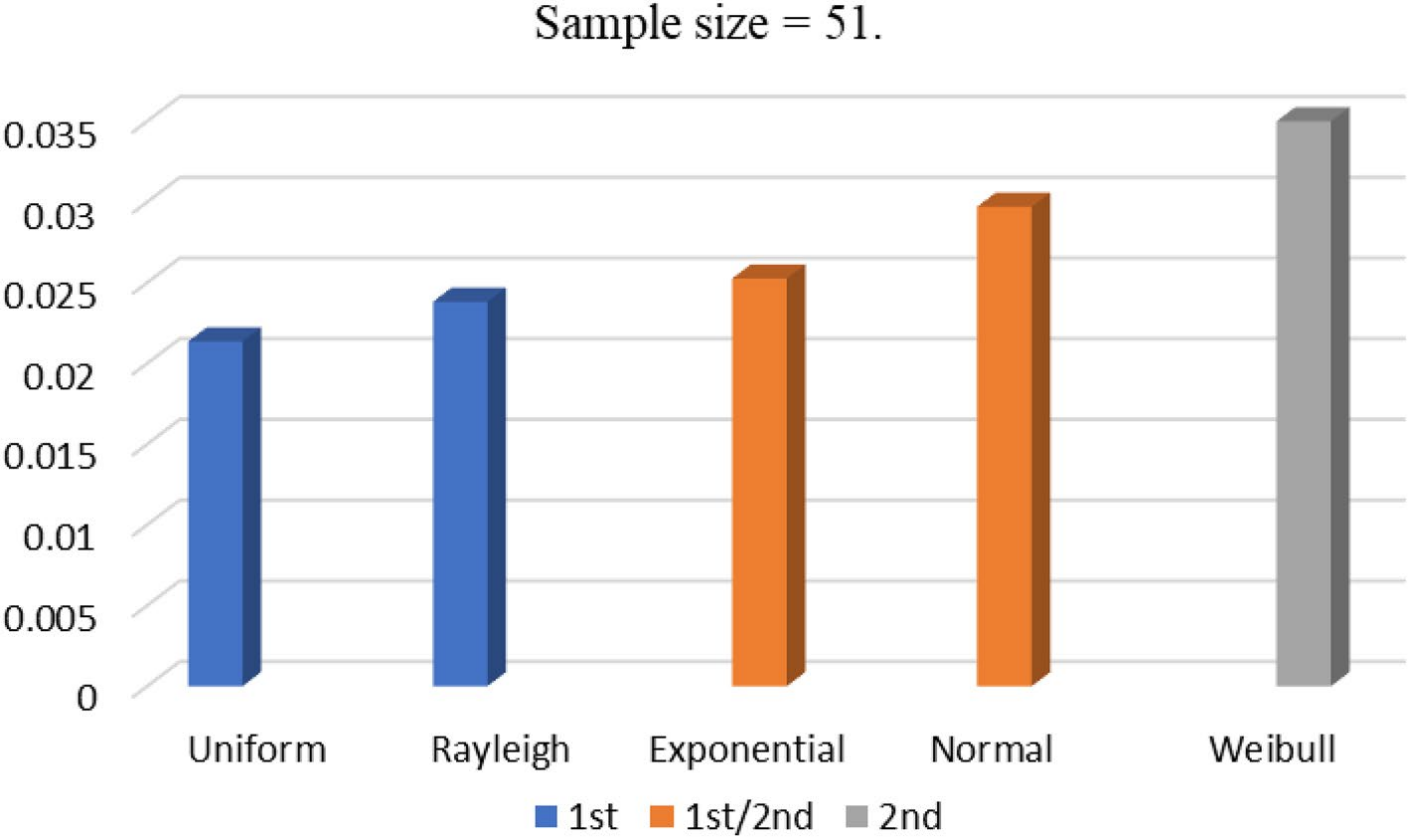
Tukey test results for different randomness via SSA for 11-level case (10th sub-scenario).

Table 1 tabulates the minimum value of the objective function provided by the APSO algorithm for a five-dimensional problem for varying values of population and maximum number of iterations. The minimum value which is provided by RR is indicated in the table. Same scenario is presented for SSA via Table 2 and Fig. 3. These results show that NR gives the overall least value for 11-level problem with SSA algorithm. In SPSS, the set significance value is 0.05 whereas the one obtained after the test for the sub-scenario of population of 2000 and 5000 maximum iterations with APSO is 0.00 and with SSA is 0.007. Hence for both algorithms, the datasets obtained with different randomizations have different means. To check where the significant difference lies, the post-hoc test of Tukey has been chosen, and the results for APSO and SSA algorithms are depicted in Figs. 4 and 5, respectively.

**Table 1:** Minimum objective values variation with randomness, population and iterations via APSO for 11-level case.

Sr #	Max iterations	Population	ER	NR	RR	UR	WR
1	500	100	2.42 × 10^−17^	1.45 × 10^−17^	5.45 × 10^−18^	6.91 × 10^−17^	1.37 × 10^−17^
2	1000	100	7.12 × 10^−18^	9.65 × 10^−18^	4.95 × 10^−19^	3.23 × 10^−18^	3.96 × 10^−17^
3	2000	100	3.24 × 10^−18^	1.83 × 10^−17^	2.73 × 10^−18^	6.39 × 10^−18^	0.00419
4	500	250	1.44 × 10^−17^	1.63 × 10^−17^	3.54 × 10^−18^	2.33 × 10^−17^	2.17 × 10^−17^
5	1000	250	3.24 × 10^−18^	1.63 × 10^−17^	**2.48 × 10**^−**19**^	3.79 × 10^−18^	2.17 × 10^−17^
6	2000	250	5.56 × 10^−18^	2.17 × 10^−17^	4.46 × 10^−18^	5.37 × 10^−18^	2.17 × 10^−17^
7	500	500	1.44 × 10^−17^	1.58 × 10^−17^	1.24 × 10^−18^	2.17 × 10^−17^	1.92 × 10^−17^
8	1000	500	4.95 × 10^−19^	4.21 × 10^−18^	2.48 × 10^−18^	4.21 × 10^−18^	0.00419
9	2000	500	3.12 × 10^−18^	0.00147	1.49 × 10^−18^	4.96 × 10^−19^	2.44 × 10^−17^
10	5000	2000	2.48 × 10^−18^	1.21 × 10^−17^	3.24 × 10^−18^	6.37 × 10^−18^	0.00419

**Table 2:** Minimum objective values variation with randomness for 11-level case with SSA (10th sub-scenario).

Max iterations	Population	ER	NR	RR	UR	WR
5000	2000	0.01186916	**0.00346284**	0.00874005	0.0099402	0.005230281

The results in Fig. 4 show that UR and RR perform better than others for APSO under the tenth sub-scenario of 11-level case. Whereas Fig. 5 shows that all perform the same under similar conditions except WR for SSA algorithm. The rest of the results are tabulated in the upcoming tables and figures.

**Figure 6:**
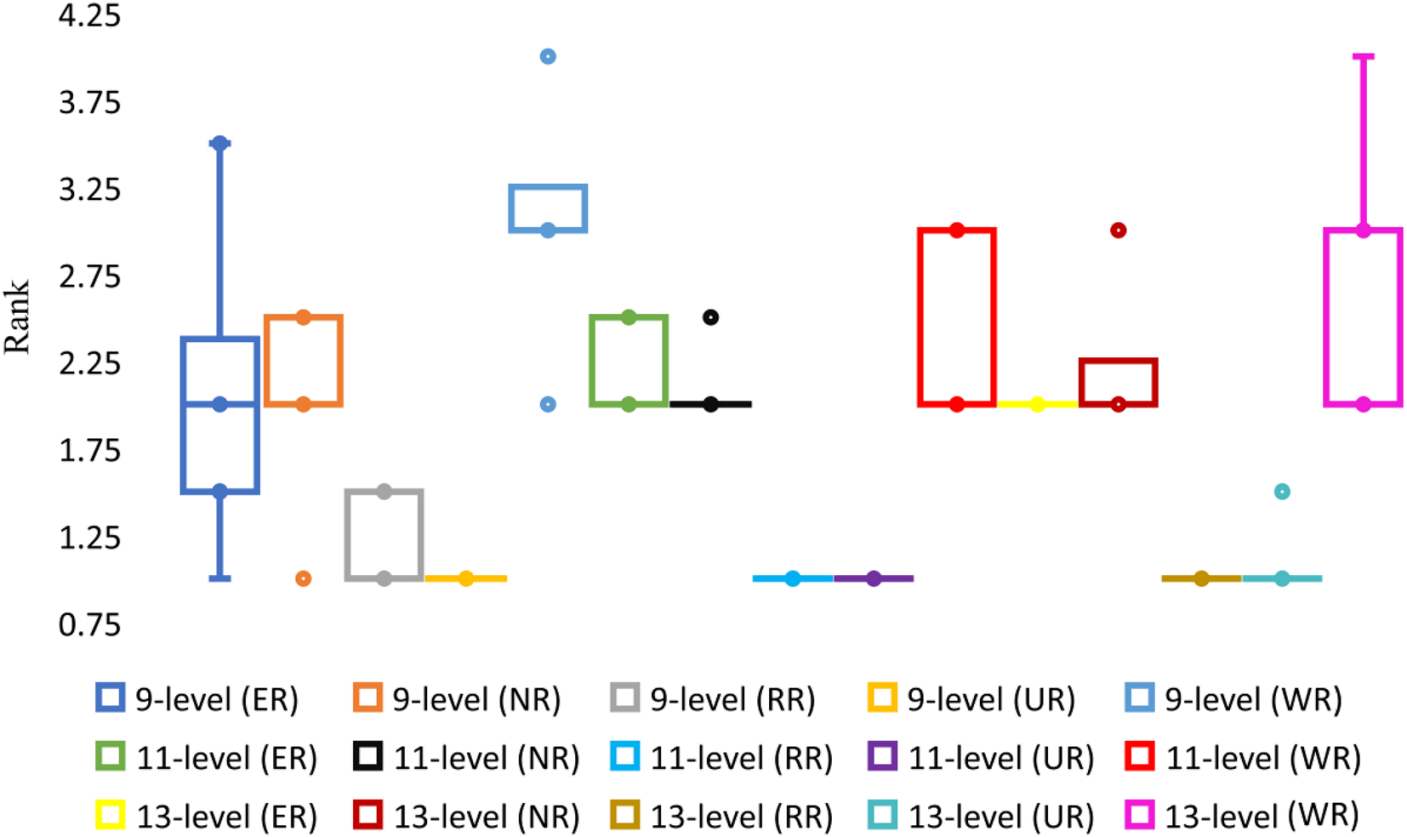
Post-hoc test results for APSO.

**Figure 7:**
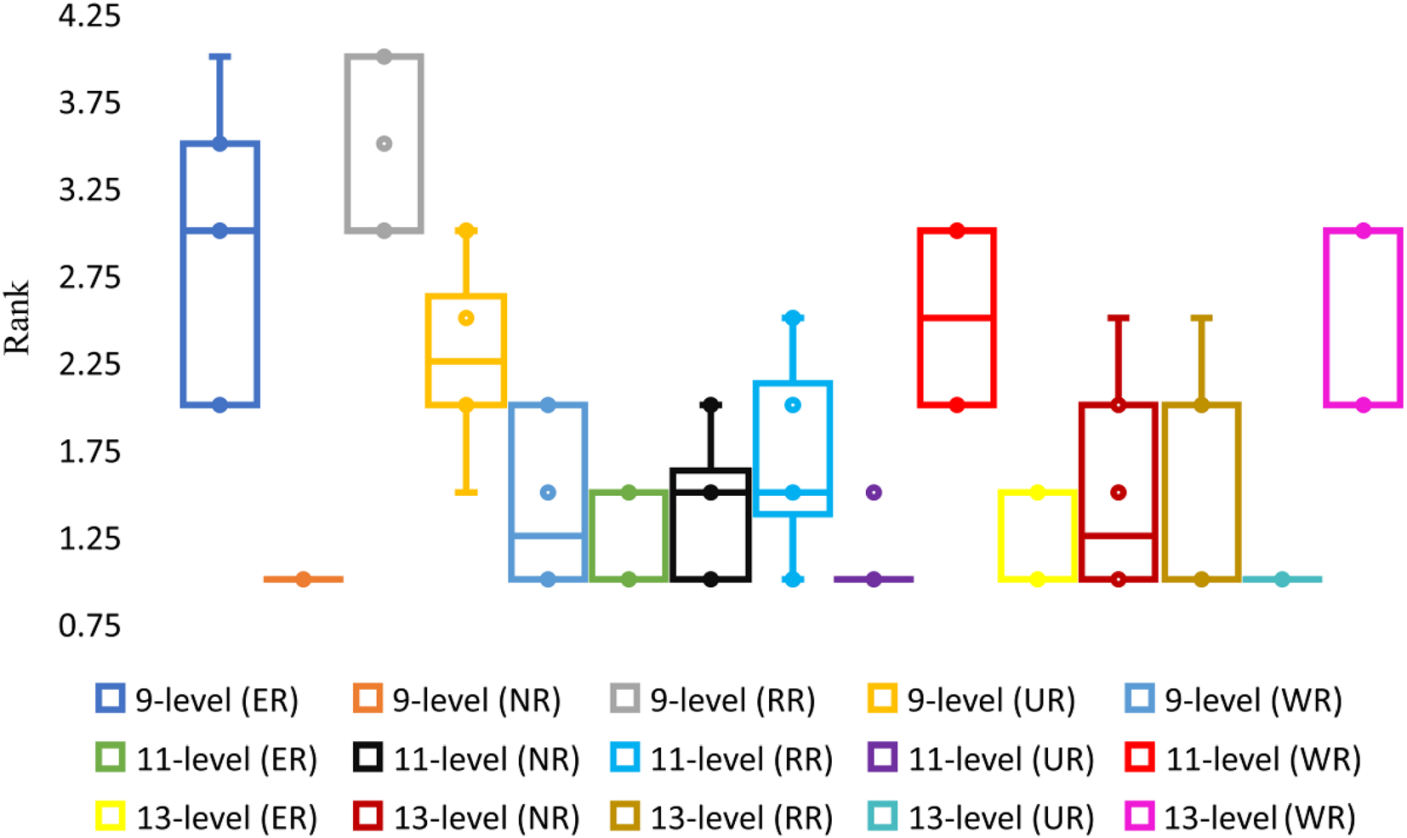
Post-hoc test results for SSA.

Table 3 shows the impact of randomizations for varying circumstances with the APSO algorithm and also tabulate the minimum value for each case with the randomization that provided such value. Figure 6 shows the ranks variation under different sub-scenarios via post-hoc test results on mean values basis for APSO. Similar stuff is tabulated in Table 4 and portrayed in Fig. 7 for SSA algorithm. All the mentioned tables and figures show that different randomizations provide different minimum and mean values. RR performed best in the case of APSO as it provided the best minimum values and first-ranked average values. In the case of SSA algorithm, although the best average values are provided by multiple randomizations, but the best minimum values are mostly provided by NR. Different randomizations perform better with different algorithms. Moreover, it is not necessary that the randomization providing better mean results will give best extreme results.

**Table 3:** Statistical comparison of the impact of randomness with APSO for varying population and iterations.

Sr #	Max itera-tions	Popula-tion	9-level case			11-level case			13-level case		
			Sig value	Minimum value	Minimum value provided by	Sig value	Minimum value	Minimum value provided by	Sig value	Minimum value	Minimum value provided by
1	500	100	0.000	2.47 × 10^−18^	RR	0.000	5.45 × 10^−18^	RR	0.000	7.91 × 10^−18^	RR
2	1000	100	0.000	0	ER, RR, UR	0.000	**4.95 × 10**^−**19**^	**RR**	0.000	5.57 × 10^−18^	RR
3	2000	100	0.000	0	ER, RR, UR	0.000	2.73 × 10^−18^	RR	0.000	4.7 × 10^−18^	RR
4	500	250	0.000	2.47 × 10^−18^	RR	0.000	3.54 × 10^−18^	RR	0.000	**1.66 × 10**^−**18**^	**RR**
5	1000	250	0.000	0	ER, RR, UR	0.000	2.48 × 10^−19^	RR	0.000	8.53 × 10^−18^	ER
6	2000	250	0.000	0	ER, RR, UR	0.000	4.46 × 10^−18^	RR	0.000	4.16 × 10^−18^	ER
7	500	500	0.000	0	RR	0.000	1.24 × 10^−18^	RR	0.000	8.85 × 10^−18^	RR
8	1000	500	0.000	0	ER, RR, UR	0.000	4.96 × 10^−19^	ER	0.000	5.69 × 10^−18^	RR
9	2000	500	0.000	0	ER, NR, RR	0.000	4.96 × 10^−19^	UR	0.000	3.58 × 10^−18^	RR
10	5000	2000	0.000	0	ER, RR, UR	0.000	2.48 × 10^−18^	ER	0.000	2.48 × 10^−18^	RR

**Table 4:** Statistical comparison of the impact of randomness with SSA for varying population and iterations.

Sr #	Max itera-tions	Popula-tion	9-level case			11-level case			13-level case		
			Sig value	Minimum value	Minimum value provided by	Sig value	Minimum value	Minimum value provided by	Sig value	Minimum value	Minimum value provided by
1	500	100	0.000	0.00466	WR	0.000	0.00849	NR	0.000	0.00879	NR
2	1000	100	0.000	0.00439	ER	0.000	0.00426	NR	0.000	0.00773	WR
3	2000	100	0.000	0.00227	WR	0.000	0.00494	WR	0.000	0.01029	RR
4	500	250	0.000	0.00327	NR	0.005	0.00782	NR	0.000	0.01148	WR
5	1000	250	0.000	0.00319	WR	0.000	0.00511	NR	0.000	0.01421	NR
6	2000	250	0.000	0.00147	WR	0.000	0.00372	NR	0.000	0.01298	ER
7	500	500	0.000	0.00439	NR	0.000	0.00603	WR	0.001	0.01265	NR
8	1000	500	0.000	0.00177	NR	0.000	0.00877	NR	0.000	0.00591	NR
9	2000	500	0.000	0.00268	NR	0.000	0.00637	NR	0.000	0.0124	NR
10	5000	2000	0.000	**0.00093**	**NR**	0.007	**0.00346**	**NR**	0.000	**0.00499**	**NR**

Finally, by using the benefits of ample data size and central limit theorem^20^, both algorithms are compared via independent t-test regarding their mean objective values. In case of lower sample size, Mann–Whitney U test^21^ can be used. The significance criterion is set at 95%. The results regarding this test are tabulated below.

**Figure 8:**
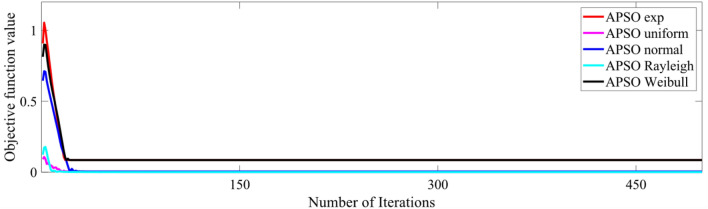
Convergence behavior of APSO algorithm with different randomizations (11-level, population = 500).

**Figure 9:**
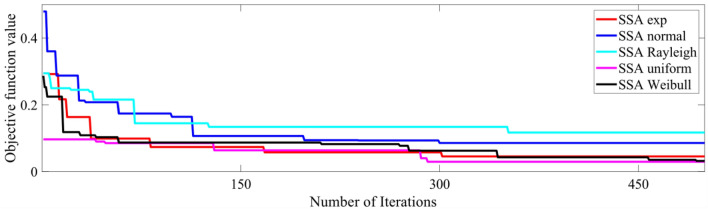
Convergence behavior of SSA algorithm with different randomizations (11-level, population = 500).

Tables 5, 6, 7, 8, and 9 show that the type of randomness impacts the independent t-test results. So, with certain type of randomness, algorithms may perform equally well. But, with some other randomness, one of those algorithms supersedes the other(s). Moreover, different algorithms may perform better under different randomness depending upon the logic behind the algorithm and its mathematical modeling. In these tables, where the significance value is less than the set value (0.05), the algorithms are proven to have different performances, and then the next column shows which algorithm gives a lesser mean value (better performance). The no-free lunch theorem also explains the different performance of algorithms when dealing with different problem statements^22^. The convergence patterns of the five different randomization-based APSO and SSA algorithms for an 11-level scenario with population and maximum iterations of 500 each are shown in Figs. 8 and 9, respectively.

**Table 5:** Statistical comparison of the impact of algorithm with exponential randomization for varying population and iterations.

9-level with ER				11-level with ER				13-level with ER			
Max itera-tions	Popul-ation	Sig value	Lesser mean value provided by	Max itera-tions	Popul-ation	Sig value	Lesser mean value provided by	Max itera-tions	Popula-tion	Sig value	Lesser mean value provided by
500	100	0.14	APSO, SSA	500	100	0.399	APSO, SSA	500	100	0.383	APSO, SSA
1000	100	0.002	SSA	1000	100	0.377	APSO, SSA	1000	100	0.182	APSO, SSA
2000	100	0.001	SSA	2000	100	0.085	APSO, SSA	2000	100	0.018	SSA
500	250	0.682	APSO, SSA	500	250	0.357	APSO, SSA	500	250	0.070	APSO, SSA
1000	250	0.067	APSO, SSA	1000	250	0.118	APSO, SSA	1000	250	0.058	APSO, SSA
2000	250	0.001	SSA	2000	250	0.064	APSO, SSA	2000	250	0.992	APSO, SSA
500	500	0.710	APSO, SSA	500	500	0.035	APSO	500	500	0.068	APSO, SSA
1000	500	0.009	SSA	1000	500	0.081	APSO, SSA	1000	500	0.036	SSA
2000	500	0.121	APSO, SSA	2000	500	0.009	SSA	2000	500	0.141	APSO, SSA
5000	2000	0.087	APSO, SSA	5000	2000	0.032	SSA	5000	2000	0.085	APSO, SSA

**Table 6:** Statistical comparison of the impact of algorithm with normal randomization for varying population and iterations.

9-level with NR				11-level with NR				13-level with NR			
Max itera-tions	Popula-tion	Sig value	Lesser mean value provided by	Max itera-tions	Popula-tion	Sig value	Lesser mean value provided by	Max itera-tions	Popula-tion	Sig value	Lesser mean value provided by
500	100	0.006	SSA	500	100	0.585	APSO, SSA	500	100	0.083	APSO, SSA
1000	100	0.353	APSO, SSA	1000	100	0.833	APSO, SSA	1000	100	0.066	APSO, SSA
2000	100	0.002	SSA	2000	100	0.503	APSO, SSA	2000	100	0.001	SSA
500	250	0.005	SSA	500	250	0.316	APSO, SSA	500	250	0.017	SSA
1000	250	0.013	SSA	1000	250	0.947	APSO, SSA	1000	250	0.001	SSA
2000	250	0.000	SSA	2000	250	0.061	APSO, SSA	2000	250	0.000	SSA
500	500	0.003	SSA	500	500	0.607	APSO, SSA	500	500	0.151	APSO, SSA
1000	500	0.005	SSA	1000	500	0.389	APSO, SSA	1000	500	0.003	SSA
2000	500	0.000	SSA	2000	500	0.000	SSA	2000	500	0.000	SSA
5000	2000	0.000	SSA	5000	2000	0.001	SSA	5000	2000	0.001	SSA

**Table 7:** Statistical comparison of the impact of algorithm with Rayleigh randomization for varying population and iterations.

9-level with RR				11-level with RR				13-level with RR			
Max itera-tions	Popula-tion	Sig value	Lesser mean value provided by	Max itera-tions	Popula-tion	Sig value	Lesser mean value provided by	Max itera-tions	Popula-tion	Sig value	Lesser mean value provided by
500	100	0.000	APSO	500	100	0.000	APSO	500	100	0.000	APSO
1000	100	0.000	APSO	1000	100	0.000	APSO	1000	100	0.000	APSO
2000	100	0.000	APSO	2000	100	0.000	APSO	2000	100	0.000	APSO
500	250	0.051	APSO, SSA	500	250	0.000	APSO	500	250	0.000	APSO
1000	250	0.000	APSO	1000	250	0.000	APSO	1000	250	0.000	APSO
2000	250	0.027	APSO	2000	250	0.000	APSO	2000	250	0.000	APSO
500	500	0.000	APSO	500	500	0.000	APSO	500	500	0.000	APSO
1000	500	0.112	APSO, SSA	1000	500	0.000	APSO	1000	500	0.000	APSO
2000	500	0.186	APSO, SSA	2000	500	0.000	APSO	2000	500	0.000	APSO
5000	2000	0.181	APSO, SSA	5000	2000	0.000	APSO	5000	2000	0.000	APSO

**Table 8:** Statistical comparison of the impact of algorithm with uniform randomization for varying population and iterations.

9-level with UR				11-level with UR				13-level with UR			
Max itera-tions	Popula-tion	Sig value	Lesser mean value provided by	Max itera-tions	Popula-tion	Sig value	Lesser mean value provided by	Max itera-tions	Popula-tion	Sig value	Lesser mean value provided by
500	100	0.000	APSO	500	100	0.000	APSO	500	100	0.000	APSO
1000	100	0.000	APSO	1000	100	0.000	APSO	1000	100	0.000	APSO
2000	100	0.000	APSO	2000	100	0.000	APSO	2000	100	0.000	APSO
500	250	0.000	APSO	500	250	0.000	APSO	500	250	0.000	APSO
1000	250	0.000	APSO	1000	250	0.000	APSO	1000	250	0.000	APSO
2000	250	0.000	APSO	2000	250	0.000	APSO	2000	250	0.000	APSO
500	500	0.000	APSO	500	500	0.000	APSO	500	500	0.000	APSO
1000	500	0.000	APSO	1000	500	0.000	APSO	1000	500	0.000	APSO
2000	500	0.000	APSO	2000	500	0.000	APSO	2000	500	0.000	APSO
5000	2000	0.000	APSO	5000	2000	0.000	APSO	5000	2000	0.000	APSO

**Table 9:** Statistical comparison of the impact of algorithm with Weibull randomization for varying population and iterations.

9-level with WR				11-level with WR				13-level with WR			
Max itera-tions	Popula-tion	Sig value	Lesser mean value provided by	Max itera-tions	Popula-tion	Sig value	Lesser mean value provided by	Max itera-tions	Popula-tion	Sig value	Lesser mean value provided by
500	100	0.000	SSA	500	100	0.478	APSO, SSA	500	100	0.001	SSA
1000	100	0.000	SSA	1000	100	0.03	SSA	1000	100	0.000	SSA
2000	100	0.000	SSA	2000	100	0.243	APSO, SSA	2000	100	0.000	SSA
500	250	0.000	SSA	500	250	0.012	SSA	500	250	0.000	SSA
1000	250	0.000	SSA	1000	250	0.054	APSO, SSA	1000	250	0.000	SSA
2000	250	0.000	SSA	2000	250	0.423	APSO, SSA	2000	250	0.000	SSA
500	500	0.000	SSA	500	500	0.025	SSA	500	500	0.003	SSA
1000	500	0.000	SSA	1000	500	0.003	SSA	1000	500	0.000	SSA
2000	500	0.000	SSA	2000	500	0.002	SSA	2000	500	0.000	SSA
5000	2000	0.000	SSA	5000	2000	0.000	SSA	5000	2000	0.000	SSA

## Conclusion and Future Works

The presented study glorifies the importance of incorporating different randomization techniques in the canonical structures of APSO and SSA algorithms to solve multiple cases of SHE. ANOVA test accompanied by Tukey post-hoc test is made the basis of the statistical scale to decide the dominance of randomization type(s) while the independent t-test is considered the basis to check the algorithm superiority. The statistical analysis shows that different randomizations impact the outcomes obtained from different algorithms differently. Mostly, the best cost values for the presented study are provided by Rayleigh randomization for APSO and by normal randomization for SSA.

The presented work covers a few types of distributions with specific values of the randomness parameters. In the future, more distributions can be incorporated with modified parameters. Moreover, combinations of initialization methods and varying randomness distributions can be deployed.

## Data Availability

The datasets used and/or analyzed during the current study are available from the corresponding author on reasonable request.
